# (3a*S*,7*S*,9a*S*,9b*R*)-3a,6,6,9a-Tetra­methyl-2-oxoperhydro­naphtho[2,1-*b*]furan-7-yl acetate

**DOI:** 10.1107/S1600536808025737

**Published:** 2008-08-16

**Authors:** Qing-Chun Huang, Bo-Gang Li, Yi-Peng Xie, Kai-Bei Yu, Guo-Lin Zhang

**Affiliations:** a Chengdu Institute of Biology, Chinese Academy of Sciences, Chengdu 610041, People’s Republic of China; b Chengdu Institute of Organic Chemistry, Chinese Academy of Sciences, Chengdu 610041, People’s Republic of China

## Abstract

The title compound (common name: 3β-acet­oxy-8-*epi*-sclareolide), C_18_H_28_O_4_, is a sclareolide derivative, which was synthesized from 9(11)-en-3β-acet­oxy-8-*epi*-sclareolide. In the mol­ecular structure, the two six-membered rings display chair conformations and the five-membered ring displays an envelope conformation. Weak inter­molecular C—H⋯O hydrogen bonding is present in the crystal structure.

## Related literature

For general background, see: Choudhary *et al.* (2004 ▶); Quideau *et al.* (2002 ▶). For related structures, see: Devi *et al.* (2004 ▶); Bhattacharyya *et al.* (2006 ▶). For synthesis, see: Yang *et al.* (2006 ▶).
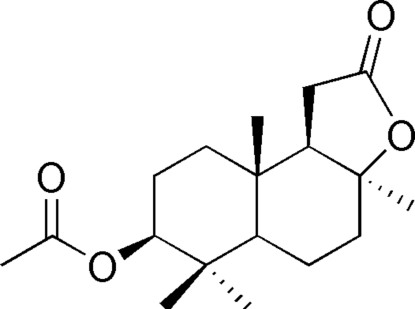


## Experimental

### 

#### Crystal data

C_18_H_28_O_4_
*M* *_r_* = 308.40
Monoclinic, 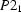

*a* = 10.1935 (5) Å
*b* = 7.3226 (3) Å
*c* = 11.3056 (4) Å
β = 99.2940 (1)°
*V* = 832.81 (6) Å^3^
*Z* = 2
Mo *K*α radiation
μ = 0.09 mm^−1^
*T* = 153 (2) K
0.60 × 0.54 × 0.47 mm

#### Data collection

Rigaku R-AXIS RAPID IP diffractometer
Absorption correction: none
8195 measured reflections
2046 independent reflections
1943 reflections with *I* > 2σ(*I*)
*R*_int_ = 0.018

#### Refinement

*R*[*F* ^2^ > 2σ(*F* ^2^)] = 0.032
*wR*(*F* ^2^) = 0.086
*S* = 0.99
2046 reflections
205 parameters
1 restraint
H-atom parameters constrained
Δρ_max_ = 0.20 e Å^−3^
Δρ_min_ = −0.14 e Å^−3^

### 

Data collection: *RAPID-AUTO* (Rigaku, 2004 ▶); cell refinement: *RAPID-AUTO*; data reduction: *RAPID-AUTO*; program(s) used to solve structure: *SHELXTL* (Sheldrick, 2008 ▶); program(s) used to refine structure: *SHELXTL*; molecular graphics: *SHELXTL*; software used to prepare material for publication: *SHELXTL*.

## Supplementary Material

Crystal structure: contains datablocks global, I. DOI: 10.1107/S1600536808025737/xu2447sup1.cif

Structure factors: contains datablocks I. DOI: 10.1107/S1600536808025737/xu2447Isup2.hkl

Additional supplementary materials:  crystallographic information; 3D view; checkCIF report

## Figures and Tables

**Table 1:** Hydrogen-bond geometry (Å, °)

*D*—H⋯*A*	*D*—H	H⋯*A*	*D*⋯*A*	*D*—H⋯*A*
C16—H16*B*⋯O2^i^	0.98	2.55	3.383 (3)	143

Symmetry code: (i) 
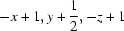
.

**Fig. 1.**
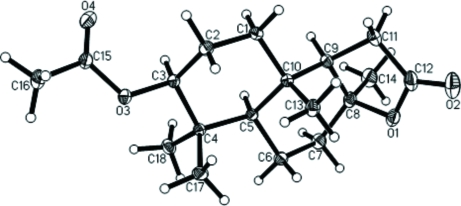
The molecular structure of the title compound, displacement ellipsoids are drawn at the 30% probability level.

## supplementary crystallographic information

### Comment

Sclareolide has exhibited phytotoxic activity and cytotoxicity against human cancer cell lines (Choudhary *et al.*, 2004). It also be important pharmaceutical intermediates (Quideau *et al.*, 2002). Here we report the synthesis and crystal structure of the title compound which is a 8-epi-sclareolide type compound.

The molecular structure is shown in Fig. 1. The molecule contains two six-membered rings, A (atoms C1–C5/C10) and B (atoms C5–C10), and one five-membered lactone rings C (C8/C9/O1/C11/C12). The cyclohexane ring A and the cyclohexane ring B exist both in chair conformation. The γ-lactone rings C adopt envelope conformations with C9 at the flap (Devi *et al.*, 2004; Bhattacharyya *et al.*, 2006). The rings A/B are *trans* fused and the rings B/C are *cis* fused. The C1/C2/C4/C5, C6/C7/C9/C10, C8/C11/C12/O1 form least square plane D, E and F, respectively. The dihedral angels between planes D and E is 15.50 (8)°, between planes E and F is 59.03 (7)°, between planes D and F is 43.54 (7)°. The C3 and C10 deviate from plane D by 0.634 (2)and 0.644 (2) Å, respectively. The C5 and C8 deviate from plane E by 0.765 (2) and 0.382 (3) Å, respectively. The C9 deviates from plane F by 0.566 (3) Å.

The intermolacular weak C—H···O hydrogen bonding presents in the crystal structure (Table 1).

### Experimental

To a methanol solution (10 ml) of 9(11)-en-3β-Acetoxy-8-epi-sclareolide (1 mmol) (Yang *et al.*, 2006) was added NiCl_2_.6H_2_O (1 mmol), and the mixture was cooled to 273 K with an ice bath, then NaBH_4_ (4 mmol) was added in small portions over 30 min. The ice bath was removed and the reaction mixture was left stirred for 4 h at room temperature. Then the suspension was filtered, and after usual workup, the residue was purified by flash chromatography eluted with petroleum ether–ethyl acetate (10:1) to afford the title compound. Yield (97%).

### Refinement

H atoms were placed in calculated positions with C—H = 0.98–1.00 Å, and refined in riding mode with *U*_iso_(H) = 1.2*U*_eq_(C). The absolute configuration has been assigned by reference to an unchanging chiral centre in the synthetic procedure; Friedel pairs were merged.

### Crystal data

**Table:**

C_18_H_28_O_4_	*F*_000_ = 336
*M**_r_* = 308.40	*D*_x_ = 1.230 Mg m^−^^3^
Monoclinic, *P*2_1_	Mo *K*α radiation λ = 0.71073 Å
Hall symbol: P 2yb	Cell parameters from 7613 reflections
*a* = 10.1935 (5) Å	θ = 3.3–27.5º
*b* = 7.3226 (3) Å	µ = 0.09 mm^−^^1^
*c* = 11.3056 (4) Å	*T* = 153 (2) K
β = 99.2940 (1)º	Block, colourless
*V* = 832.81 (6) Å^3^	0.60 × 0.54 × 0.47 mm
*Z* = 2	

### Data collection

**Table:**

Rigaku R-AXIS RAPID IP diffractometer	1943 reflections with *I* > 2σ(*I*)
Radiation source: Rotating Anode	*R*_int_ = 0.018
Monochromator: graphite	θ_max_ = 27.5º
*T* = 153(2) K	θ_min_ = 3.3º
ω scans	*h* = −13→13
Absorption correction: none	*k* = −9→9
8195 measured reflections	*l* = −14→12
2046 independent reflections	

### Refinement

**Table:**

Refinement on *F*^2^	Secondary atom site location: difference Fourier map
Least-squares matrix: full	Hydrogen site location: inferred from neighbouring sites
*R*[*F*^2^ > 2σ(*F*^2^)] = 0.032	H-atom parameters constrained
*wR*(*F*^2^) = 0.086	*w* = 1/[σ^2^(*F*_o_^2^) + (0.0526*P*)^2^ + 0.1209*P*] where *P* = (*F*_o_^2^ + 2*F*_c_^2^)/3
*S* = 0.99	(Δ/σ)_max_ < 0.001
2046 reflections	Δρ_max_ = 0.20 e Å^−^^3^
205 parameters	Δρ_min_ = −0.14 e Å^−^^3^
1 restraint	Extinction correction: SHELXTL (Sheldrick, 2008), Fc^*^=kFc[1+0.001xFc^2^λ^3^/sin(2θ)]^-1/4^
Primary atom site location: structure-invariant direct methods	Extinction coefficient: 0.065 (6)

### Special details

**Table:**

Geometry. All e.s.d.'s (except the e.s.d. in the dihedral angle between two l.s. planes) are estimated using the full covariance matrix. The cell e.s.d.'s are taken into account individually in the estimation of e.s.d.'s in distances, angles and torsion angles; correlations between e.s.d.'s in cell parameters are only used when they are defined by crystal symmetry. An approximate (isotropic) treatment of cell e.s.d.'s is used for estimating e.s.d.'s involving l.s. planes.
Refinement. Refinement of *F*^2^ against ALL reflections. The weighted *R*-factor *wR* and goodness of fit *S* are based on *F*^2^, conventional *R*-factors *R* are based on *F*, with *F* set to zero for negative *F*^2^. The threshold expression of *F*^2^ > σ(*F*^2^) is used only for calculating *R*-factors(gt) *etc*. and is not relevant to the choice of reflections for refinement. *R*-factors based on *F*^2^ are statistically about twice as large as those based on *F*, and *R*- factors based on ALL data will be even larger.

### Fractional atomic coordinates and isotropic or equivalent isotropic displacement parameters (Å^2^)

**Table:**

	*x*	*y*	*z*	*U*_iso_*/*U*_eq_	
O1	0.15453 (13)	0.2174 (2)	0.55280 (13)	0.0477 (4)	
O2	0.10361 (18)	0.3434 (4)	0.37252 (14)	0.0809 (6)	
O3	0.78754 (11)	0.58869 (17)	0.86839 (11)	0.0377 (3)	
O4	0.77677 (15)	0.8581 (2)	0.96045 (15)	0.0599 (4)	
C1	0.44238 (16)	0.6709 (2)	0.70097 (15)	0.0344 (3)	
H1A	0.4104	0.7464	0.6295	0.041*	
H1B	0.4025	0.7199	0.7687	0.041*	
C2	0.59341 (16)	0.6859 (2)	0.73086 (15)	0.0370 (4)	
H2A	0.6341	0.6395	0.6630	0.044*	
H2B	0.6194	0.8154	0.7443	0.044*	
C3	0.64282 (15)	0.5757 (2)	0.84229 (14)	0.0301 (3)	
H3	0.6051	0.6290	0.9110	0.036*	
C4	0.60784 (14)	0.3708 (2)	0.83267 (13)	0.0280 (3)	
C5	0.45489 (14)	0.3533 (2)	0.78539 (13)	0.0259 (3)	
H5	0.4102	0.3959	0.8529	0.031*	
C6	0.40994 (16)	0.1550 (2)	0.76243 (16)	0.0360 (4)	
H6A	0.4350	0.1121	0.6861	0.043*	
H6B	0.4551	0.0762	0.8277	0.043*	
C7	0.25934 (17)	0.1404 (3)	0.75616 (17)	0.0427 (4)	
H7A	0.2310	0.0175	0.7257	0.051*	
H7B	0.2388	0.1502	0.8386	0.051*	
C8	0.17711 (15)	0.2812 (3)	0.67859 (15)	0.0386 (4)	
C9	0.24142 (15)	0.4709 (3)	0.66974 (15)	0.0345 (3)	
H9	0.2175	0.5507	0.7348	0.041*	
C10	0.39595 (14)	0.4729 (2)	0.67643 (13)	0.0275 (3)	
C11	0.1657 (2)	0.5373 (3)	0.54912 (19)	0.0482 (5)	
H11A	0.0824	0.6003	0.5595	0.058*	
H11B	0.2210	0.6213	0.5093	0.058*	
C12	0.13711 (18)	0.3637 (4)	0.47870 (18)	0.0523 (5)	
C13	0.43749 (17)	0.4084 (3)	0.55821 (14)	0.0384 (4)	
H13A	0.5347	0.4090	0.5663	0.046*	
H13B	0.4000	0.4909	0.4932	0.046*	
H13C	0.4043	0.2844	0.5399	0.046*	
C14	0.03976 (18)	0.2954 (4)	0.7163 (2)	0.0566 (6)	
H14A	−0.0028	0.1750	0.7102	0.068*	
H14B	−0.0153	0.3816	0.6635	0.068*	
H14C	0.0494	0.3388	0.7993	0.068*	
C15	0.84029 (17)	0.7424 (3)	0.92062 (16)	0.0399 (4)	
C16	0.98747 (19)	0.7482 (3)	0.9221 (2)	0.0523 (5)	
H16A	1.0279	0.8355	0.9830	0.063*	
H16B	1.0056	0.7864	0.8432	0.063*	
H16C	1.0252	0.6266	0.9411	0.063*	
C17	0.69680 (16)	0.2695 (3)	0.75667 (16)	0.0395 (4)	
H17A	0.6594	0.1485	0.7353	0.047*	
H17B	0.7863	0.2562	0.8028	0.047*	
H17C	0.7014	0.3393	0.6835	0.047*	
C18	0.63524 (17)	0.2918 (3)	0.96055 (16)	0.0410 (4)	
H18A	0.7252	0.3255	0.9987	0.049*	
H18B	0.6274	0.1585	0.9569	0.049*	
H18C	0.5705	0.3412	1.0074	0.049*	

### Atomic displacement parameters (Å^2^)

**Table:**

	*U*^11^	*U*^22^	*U*^33^	*U*^12^	*U*^13^	*U*^23^
O1	0.0391 (7)	0.0550 (9)	0.0468 (7)	−0.0076 (6)	0.0003 (5)	−0.0144 (6)
O2	0.0773 (11)	0.1122 (17)	0.0453 (8)	−0.0063 (12)	−0.0140 (7)	−0.0085 (11)
O3	0.0267 (6)	0.0346 (6)	0.0499 (7)	−0.0043 (5)	0.0006 (5)	−0.0051 (5)
O4	0.0517 (8)	0.0469 (8)	0.0773 (10)	−0.0032 (7)	−0.0014 (7)	−0.0222 (8)
C1	0.0347 (8)	0.0271 (7)	0.0390 (8)	0.0021 (7)	−0.0014 (6)	0.0043 (6)
C2	0.0352 (8)	0.0283 (8)	0.0455 (9)	−0.0064 (7)	0.0008 (6)	0.0055 (7)
C3	0.0243 (7)	0.0283 (8)	0.0365 (8)	−0.0006 (6)	0.0017 (5)	−0.0027 (6)
C4	0.0223 (6)	0.0265 (7)	0.0347 (7)	0.0003 (6)	0.0027 (5)	−0.0008 (6)
C5	0.0233 (6)	0.0255 (7)	0.0291 (7)	0.0001 (5)	0.0050 (5)	−0.0019 (6)
C6	0.0320 (8)	0.0273 (8)	0.0477 (9)	−0.0029 (7)	0.0039 (7)	0.0012 (7)
C7	0.0347 (9)	0.0406 (10)	0.0529 (10)	−0.0109 (8)	0.0072 (7)	0.0021 (8)
C8	0.0267 (7)	0.0466 (10)	0.0418 (9)	−0.0057 (7)	0.0039 (6)	−0.0079 (8)
C9	0.0268 (7)	0.0371 (9)	0.0382 (8)	0.0044 (7)	0.0006 (6)	−0.0045 (7)
C10	0.0267 (6)	0.0274 (7)	0.0278 (7)	0.0006 (6)	0.0026 (5)	−0.0010 (6)
C11	0.0353 (9)	0.0544 (12)	0.0501 (11)	0.0060 (8)	−0.0077 (8)	0.0034 (9)
C12	0.0358 (9)	0.0719 (14)	0.0454 (10)	−0.0014 (10)	−0.0050 (7)	−0.0045 (11)
C13	0.0367 (8)	0.0486 (10)	0.0301 (7)	−0.0023 (7)	0.0060 (6)	−0.0045 (7)
C14	0.0300 (8)	0.0750 (15)	0.0665 (12)	−0.0064 (10)	0.0132 (8)	−0.0086 (12)
C15	0.0383 (8)	0.0364 (9)	0.0415 (8)	−0.0063 (7)	−0.0041 (7)	0.0003 (8)
C16	0.0373 (9)	0.0492 (11)	0.0660 (12)	−0.0129 (9)	−0.0051 (8)	−0.0014 (10)
C17	0.0290 (7)	0.0357 (9)	0.0551 (10)	0.0030 (7)	0.0107 (7)	−0.0073 (8)
C18	0.0372 (8)	0.0409 (9)	0.0419 (9)	0.0002 (7)	−0.0021 (6)	0.0117 (7)

### Geometric parameters (Å, °)

**Table:**

O1—C12	1.354 (3)	C7—H7B	0.9900
O1—C8	1.479 (2)	C8—C14	1.532 (2)
O2—C12	1.203 (2)	C8—C9	1.546 (3)
O3—C15	1.343 (2)	C9—C11	1.534 (2)
O3—C3	1.4601 (18)	C9—C10	1.565 (2)
O4—C15	1.197 (3)	C9—H9	1.0000
C1—C2	1.526 (2)	C10—C13	1.540 (2)
C1—C10	1.536 (2)	C11—C12	1.504 (3)
C1—H1A	0.9900	C11—H11A	0.9900
C1—H1B	0.9900	C11—H11B	0.9900
C2—C3	1.512 (2)	C13—H13A	0.9800
C2—H2A	0.9900	C13—H13B	0.9800
C2—H2B	0.9900	C13—H13C	0.9800
C3—C4	1.542 (2)	C14—H14A	0.9800
C3—H3	1.0000	C14—H14B	0.9800
C4—C17	1.537 (2)	C14—H14C	0.9800
C4—C18	1.540 (2)	C15—C16	1.498 (2)
C4—C5	1.5696 (18)	C16—H16A	0.9800
C5—C6	1.532 (2)	C16—H16B	0.9800
C5—C10	1.551 (2)	C16—H16C	0.9800
C5—H5	1.0000	C17—H17A	0.9800
C6—C7	1.529 (2)	C17—H17B	0.9800
C6—H6A	0.9900	C17—H17C	0.9800
C6—H6B	0.9900	C18—H18A	0.9800
C7—C8	1.516 (3)	C18—H18B	0.9800
C7—H7A	0.9900	C18—H18C	0.9800
			
C12—O1—C8	109.23 (16)	C8—C9—C10	116.08 (13)
C15—O3—C3	117.60 (13)	C11—C9—H9	108.8
C2—C1—C10	112.21 (13)	C8—C9—H9	108.8
C2—C1—H1A	109.2	C10—C9—H9	108.8
C10—C1—H1A	109.2	C1—C10—C13	109.04 (14)
C2—C1—H1B	109.2	C1—C10—C5	108.73 (11)
C10—C1—H1B	109.2	C13—C10—C5	112.98 (13)
H1A—C1—H1B	107.9	C1—C10—C9	107.23 (13)
C3—C2—C1	109.55 (13)	C13—C10—C9	111.77 (12)
C3—C2—H2A	109.8	C5—C10—C9	106.90 (12)
C1—C2—H2A	109.8	C12—C11—C9	103.26 (17)
C3—C2—H2B	109.8	C12—C11—H11A	111.1
C1—C2—H2B	109.8	C9—C11—H11A	111.1
H2A—C2—H2B	108.2	C12—C11—H11B	111.1
O3—C3—C2	108.86 (13)	C9—C11—H11B	111.1
O3—C3—C4	106.99 (12)	H11A—C11—H11B	109.1
C2—C3—C4	114.73 (13)	O2—C12—O1	120.5 (2)
O3—C3—H3	108.7	O2—C12—C11	129.2 (3)
C2—C3—H3	108.7	O1—C12—C11	110.28 (15)
C4—C3—H3	108.7	C10—C13—H13A	109.5
C17—C4—C18	108.03 (14)	C10—C13—H13B	109.5
C17—C4—C3	111.00 (13)	H13A—C13—H13B	109.5
C18—C4—C3	107.13 (13)	C10—C13—H13C	109.5
C17—C4—C5	114.41 (13)	H13A—C13—H13C	109.5
C18—C4—C5	107.97 (12)	H13B—C13—H13C	109.5
C3—C4—C5	108.02 (12)	C8—C14—H14A	109.5
C6—C5—C10	109.50 (12)	C8—C14—H14B	109.5
C6—C5—C4	112.88 (12)	H14A—C14—H14B	109.5
C10—C5—C4	117.40 (12)	C8—C14—H14C	109.5
C6—C5—H5	105.3	H14A—C14—H14C	109.5
C10—C5—H5	105.3	H14B—C14—H14C	109.5
C4—C5—H5	105.3	O4—C15—O3	123.78 (16)
C7—C6—C5	110.17 (14)	O4—C15—C16	125.24 (18)
C7—C6—H6A	109.6	O3—C15—C16	110.98 (17)
C5—C6—H6A	109.6	C15—C16—H16A	109.5
C7—C6—H6B	109.6	C15—C16—H16B	109.5
C5—C6—H6B	109.6	H16A—C16—H16B	109.5
H6A—C6—H6B	108.1	C15—C16—H16C	109.5
C8—C7—C6	115.88 (15)	H16A—C16—H16C	109.5
C8—C7—H7A	108.3	H16B—C16—H16C	109.5
C6—C7—H7A	108.3	C4—C17—H17A	109.5
C8—C7—H7B	108.3	C4—C17—H17B	109.5
C6—C7—H7B	108.3	H17A—C17—H17B	109.5
H7A—C7—H7B	107.4	C4—C17—H17C	109.5
O1—C8—C7	109.08 (16)	H17A—C17—H17C	109.5
O1—C8—C14	106.37 (14)	H17B—C17—H17C	109.5
C7—C8—C14	109.27 (17)	C4—C18—H18A	109.5
O1—C8—C9	102.92 (14)	C4—C18—H18B	109.5
C7—C8—C9	116.62 (13)	H18A—C18—H18B	109.5
C14—C8—C9	111.92 (17)	C4—C18—H18C	109.5
C11—C9—C8	100.65 (14)	H18A—C18—H18C	109.5
C11—C9—C10	113.45 (15)	H18B—C18—H18C	109.5
			
C10—C1—C2—C3	−60.61 (18)	C14—C8—C9—C11	−77.70 (18)
C15—O3—C3—C2	77.96 (18)	O1—C8—C9—C10	−86.75 (15)
C15—O3—C3—C4	−157.52 (14)	C7—C8—C9—C10	32.6 (2)
C1—C2—C3—O3	178.97 (13)	C14—C8—C9—C10	159.43 (15)
C1—C2—C3—C4	59.15 (18)	C2—C1—C10—C13	−68.69 (16)
O3—C3—C4—C17	−45.26 (17)	C2—C1—C10—C5	54.89 (17)
C2—C3—C4—C17	75.59 (16)	C2—C1—C10—C9	170.13 (13)
O3—C3—C4—C18	72.47 (15)	C6—C5—C10—C1	−179.85 (13)
C2—C3—C4—C18	−166.68 (13)	C4—C5—C10—C1	−49.46 (17)
O3—C3—C4—C5	−171.45 (11)	C6—C5—C10—C13	−58.66 (16)
C2—C3—C4—C5	−50.60 (17)	C4—C5—C10—C13	71.73 (17)
C17—C4—C5—C6	51.22 (18)	C6—C5—C10—C9	64.69 (15)
C18—C4—C5—C6	−69.07 (17)	C4—C5—C10—C9	−164.91 (13)
C3—C4—C5—C6	175.39 (13)	C11—C9—C10—C1	79.44 (17)
C17—C4—C5—C10	−77.59 (18)	C8—C9—C10—C1	−164.68 (14)
C18—C4—C5—C10	162.11 (14)	C11—C9—C10—C13	−40.0 (2)
C3—C4—C5—C10	46.57 (17)	C8—C9—C10—C13	75.88 (18)
C10—C5—C6—C7	−65.31 (17)	C11—C9—C10—C5	−164.10 (15)
C4—C5—C6—C7	161.90 (13)	C8—C9—C10—C5	−48.22 (17)
C5—C6—C7—C8	47.1 (2)	C8—C9—C11—C12	−31.47 (18)
C12—O1—C8—C7	−152.81 (14)	C10—C9—C11—C12	93.22 (18)
C12—O1—C8—C14	89.45 (19)	C8—O1—C12—O2	−172.27 (18)
C12—O1—C8—C9	−28.36 (17)	C8—O1—C12—C11	7.91 (19)
C6—C7—C8—O1	84.82 (18)	C9—C11—C12—O2	−163.8 (2)
C6—C7—C8—C14	−159.28 (17)	C9—C11—C12—O1	16.0 (2)
C6—C7—C8—C9	−31.1 (2)	C3—O3—C15—O4	9.9 (3)
O1—C8—C9—C11	36.12 (16)	C3—O3—C15—C16	−170.25 (14)
C7—C8—C9—C11	155.46 (16)		

### Hydrogen-bond geometry (Å, °)

**Table:**

*D*—H···*A*	*D*—H	H···*A*	*D*···*A*	*D*—H···*A*
C16—H16B···O2^i^	0.98	2.55	3.383 (3)	143

Symmetry codes: (i) −*x*+1, *y*+1/2, −*z*+1.
